# Investing in the health workforce, protecting mental health: policy and action taken by WHO: Tomas Zapata

**DOI:** 10.1093/eurpub/ckac129.040

**Published:** 2022-10-25

**Authors:** T Zapata, M Langins

**Affiliations:** WHO Regional Office for Europe, Copenhagen, Denmark

## Abstract

**Background:**

The COVID-19 pandemic and the current war in Ukraine have presented opportunities to increase national leadership on mental health, including support health and care workers. Member States across the region have worked closely with the WHO to strengthen their policy levers to support the mental health and occupational safety policies for the workforce at various levels including government, organization and local service levels. To complement these policies, the WHO will launch the WHO Global Health and Care Compact at the Seventy-fifth World Health Assembly in May 2022.

**Methods:**

The WHO has convened Member States to discuss the policy levers that can support the mental health of health and care workers. These include online multistakeholder webinar series, national policy dialogs, ministerial discussions during the Regional Committee, preparatory consultations for the World Health Assembly to discuss priority areas and policy. More recently with the crisis in Ukraine, the WHO has been working closely with the UN Interagency Steering Committee (IASC) Reference Group on Mental Health and Psychosocial Support in Emergency Settings rolling out services for refugees, including health workers in these settings.

**Results:**

A number of important policy recommendations are emerging from this work: (1) strengthening national and political leadership; (2) adopting stepped approaches; (3) facilitating collaboration across professions, sectors and levels of syste for more effective responses; (4) strengthening capacity and expertise; (5) monitoring effectiveness for continuous improvement; (6) ensuring acceptability and accessibility.

**Conclusions:**

Promoting health and well-being and mental health support to the health and care workforce has become a key priority for health systems to enable sustainable national health workforces. WHO will prioritize country support on this area and the development of regional guidance.

